# Metal Oxide Nanoparticles in Therapeutic Regulation of Macrophage Functions

**DOI:** 10.3390/nano9111631

**Published:** 2019-11-16

**Authors:** Marina S. Dukhinova, Artur. Y. Prilepskii, Alexander A. Shtil, Vladimir V. Vinogradov

**Affiliations:** 1 ITMO University, Saint-Petersburg 197101, Russia; marina_dukhinova@mail.ru (M.S.D.); prilepskii@gmail.com (A.Y.P.); shtil@scamt-itmo.ru (A.A.S.); 2 Blokhin National Medical Center of Oncology, Moscow 115478, Russia

**Keywords:** nanoparticles, metal oxides, macrophages, inflammation, signal transduction, immunotherapy

## Abstract

Macrophages are components of the innate immune system that control a plethora of biological processes. Macrophages can be activated towards pro-inflammatory (M1) or anti-inflammatory (M2) phenotypes depending on the cue; however, polarization may be altered in bacterial and viral infections, cancer, or autoimmune diseases. Metal (zinc, iron, titanium, copper, etc.) oxide nanoparticles are widely used in therapeutic applications as drugs, nanocarriers, and diagnostic tools. Macrophages can recognize and engulf nanoparticles, while the influence of macrophage-nanoparticle interaction on cell polarization remains unclear. In this review, we summarize the molecular mechanisms that drive macrophage activation phenotypes and functions upon interaction with nanoparticles in an inflammatory microenvironment. The manifold effects of metal oxide nanoparticles on macrophages depend on the type of metal and the route of synthesis. While largely considered as drug transporters, metal oxide nanoparticles nevertheless have an immunotherapeutic potential, as they can evoke pro- or anti-inflammatory effects on macrophages and become essential for macrophage profiling in cancer, wound healing, infections, and autoimmunity.

## 1. Introduction

Macrophages (MФs) are the essential components of innate immunity. These active phagocytes are the first encounter for external substances, including nanoparticles (NPs). The so-called industrial NPs enter our organism in a non-specific way, as they can be delivered via inhalation, food or water intake, and skin exposure. The emerging medical applications of NPs, in particular, metal oxide NPs (MONPs), raise new questions regarding mechanisms, immunological aspects, and therapeutic relevance of MФ-NP interaction in a wide variety of physiological and pathological situations. It is well known that MФs respond to various stimuli and obtain distinct functional profiles to shape the functions of innate and adaptive immune cells. However, the mechanisms and outcomes of MФ-NP interaction remain unclear.

## 2. Metal Oxide Nanoparticles: a General Overview

### 2.1. Synthesis of Metal Oxide Nanoparticles

Presently, there are numerous synthetic procedures to obtain MONPs of almost any shape, size, and surface structure. There are three major types of MONP synthesis: physical (laser ablation, ultrasonication, spray pyrolysis, vaporization), chemical (sol-gel, hydrothermal, co-precipitation), and biological. The selected method determines the physicochemical characteristics of MONPs and the type of defects, morphology, and crystal structure [1]. Although the large-scale production of many kinds of MONPs is questionable and not a major contributor to environmental pollution, the bio-inspired, or green synthesis methods attract growing attention. These methods usually involve “wet” chemical synthesis in aqueous, ethanol, or other types of extracts obtained from plants, fungi, bacteria, or algae. Also, the major difference between the use of harsh/hazardous chemicals and extracts is that the obtained MONPs are generally functionalized by phytochemicals and are, therefore, biocompatible [2]. However, the yield and monodispersity of bio-prepared NPs, as well as reproducibility, are insufficient, due to differential concentrations of active compounds in raw material [3]. Also, mechanisms of “green” MONPs formation remain poorly investigated and deserve a more detailed analysis [4].

### 2.2. Variability of Metal Oxide Nanoparticles

Currently, MONPs of almost 30 different chemical elements are described [5]. Among the most common are alumina [6,7], cerium [8], cobalt [9], copper [10], iron [11,12], gadolinium [13], hafnium [14], magnesium [15], manganese [16], silica, titanium [17], and zinc [18] MONPs.

Cerium oxide has emerged as a “hot” topic in nanobiomedicine [19]. Cerium oxide NPs (nanoceria) have been showed to provide neuroprotective [20], antioxidant [21], antibacterial [22] effects. These materials can be synthesized by a variety of methods, including chemical fabrication via oxidation of cerium (III) ions by apoferritin [23], or green chemicals, such as leaf extracts of *Gloriosa superba L.* [24] or *Acalypha indica* [25].

Magnesium oxide NPs for antimicrobial and anticancer applications have been obtained by reduction from magnesium nitrate using bioactive compounds from algae *Sophora wightii* [26]. The aqueous extract of *Aspalathus linearis,* commonly known as rooibos, has been used to reduce palladium and nickel from palladium (II) chloride and nickel (II) nitrate hexahydrate to form PdO and NiO NPs [27].

Zinc oxide NPs are widely used as antimicrobial agents [28,29]. Contrary to previously described synthetic procedures, general methods for ZnO NPs preparation are the mechanochemical processing and physical vapor synthesis. Mechano-assisted methods are conducted in a ball mill by mixing zinc chloride with sodium carbonate following by heat treatment [30]. In PVC methods, a solid precursor is evaporated by plasma arc and then cooled and condensed in a controlled manner to obtain NPs [31]. Titanium dioxide and zinc oxide NPs are common ingredients in many commercially available cosmetics, such as sunscreens. The way of formation of these NPs is unknown, but in individual studies, their properties have been addressed, which will help to estimate their possible toxicity [32].

Iron oxide NPs (mostly, magnetite) have been approved by FDA and EMA for drug delivery [33], hyperthermia [34], or as a stand-alone drug [35]. A plethora of methods, as well as application strategies, have been comprehensively described in a recent review on magnetite NPs [36].

Copper and cobalt are the microelements essential for plant growth [37]. Recently, copper and cobalt oxide NP powders have been synthesized [38,39]. However, the fate of these NPs and their impact on consumers remain to be investigated. In these studies, NPs were obtained by wire electric explosion in an inert atmosphere under low pressure, which results in a pure metal oxide shell on the surface of NPs. In general, the presence of NPs in the soil can be a major problem and may lead to their accumulation in food crops, livestock, and in humans [40].

### 2.3. Stabilization of Metal Oxide Nanoparticles in a Biological Microenvironment

Different polymers, polyelectrolytes, or proteins are often used to stabilize the prepared NPs. These modifications can seriously alter biodistribution and toxicity. Bovine or human serum albumins can be used to functionalize both the synthesized NPs [41] or during synthesis [42]. However, even these native proteins can provoke an undesired immune response due to protein misfolding upon their binding to the NP surface. Other commonly used stabilization agents, such as polyethyleneimine (PEI) and poly(lactide-co-glycolide) (PLGA), can also be responsible for MФ activation [43]. Furthermore, surface coating with serum proteins (i.e., protein corona effect) is known to alter immunogenic properties and clearance of NPs [44]. Considering the fact that protein corona largely depends on size, surface charge, and shape of NPs, its chemical composition is not important [45].

Not only single-metal NPs but also polymetallic NPs like ZnMgO have been reported [1]. The latter materials showed a lesser tendency to aggregate in biological fluids and an increased antibacterial activity [1]. Complexes of MONPs with metal-organic frames have been described for gas storages and separators, catalysis platforms, sensors, and drug delivery platforms [46,47]. Thus, MONPs can be synthesized by a variety of methods. Regardless of this variability, MONPs enter the body through the lungs or with food, or as drugs and primarily interact with the immune system.

## 3. Macrophage Polarization as an Essential Response for Altered Cell Microenvironment

**Figure 1:**
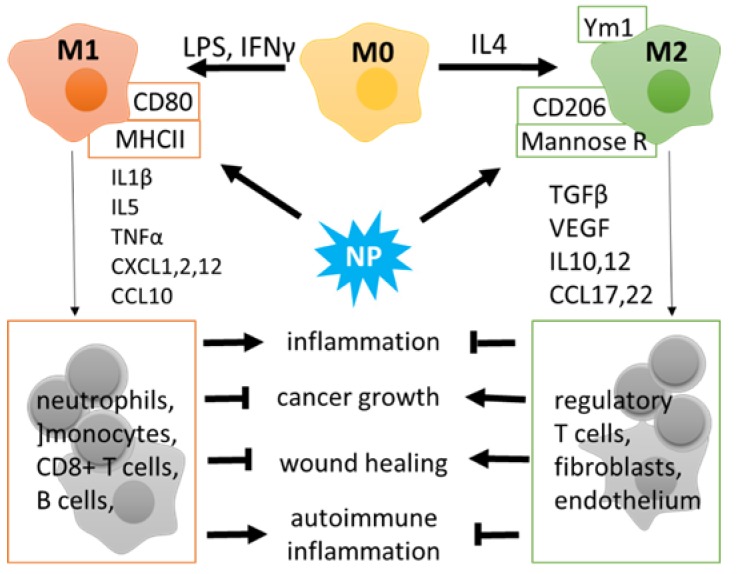
Macrophage polarization: M1 (classical, pro-inflammatory) and M2 (alternative, anti-inflammatory). M1 polarization can be triggered by lipopolysaccharides (LPS) and/or interferon gamma (INFγ). M1 macrophages express high CD80 and MHCII and produce pro-inflammatory cytokines to stimulate the innate and adaptive immune activity of monocytes, neutrophils, T- and B-lymphocytes. M2 cells are characterized by surface markers Ym1, CD206, and mannose receptor, as well as by cytokines that have a potential for immunosuppression and tissue regeneration. Tumor-associated macrophages develop an M2 phenotype and promote the immune escape of tumor cells.

MФs are a heterogeneous cell population of the myeloid lineage that exhibits phagocytic activity and participates in innate and adaptive immune reactions. MФ populations include blood-circulating monocytes derived from the bone marrow in adult mammals and tissue-resident MФs that have exclusive routes of embryonic development and may also arise from mononuclear cells that populate the organs. Resident MФs are found in all tissues, with the examples including alveolar MФs, liver MФs named Kupffer cells, brain resident microglia, etc. [48]. Major local or systemic changes in the organism, such as microbial or protozoan pathogens, trauma, or tumor growth, cause activation and infiltration of blood monocytes and polarization of tissue-resident MФs. Activated MФs are commonly divided into two subsets, that is, the classical (M1; pro-inflammatory) and alternative (M2; anti-inflammatory) (Figure 1).

M1-like MФs are characterized by the ability to release pro-inflammatory (interleukin 1 beta, Il1β, TNFα) and chemoattractant (CXCL3, -8, -10) cytokines and play an essential role in the elimination of pathogens, damaged or transformed cells, and recruitment of other immune cells to the pathological site [49,50]. However, M1 cells can also promote a cytotoxic effect in a prolonged inflammation: harming normal cells by mistake and attracting CD8+ T and B lymphocytes to attack the surrounding tissues in neurodegeneration or autoimmunity (Figure 1). M2-like MФs produce anti-inflammatory molecules and growth factors (Il10, TGFβ, VEGF) to control immunity and promote regeneration. At the same time, M2 profiling correlates with a poor prognosis in cancer and infections [51]. The division into pro- and anti-inflammatory subsets reflects the major functional activity of MФs; however, in vivo some stimuli drive MФ polarization towards different directions; these routes can be modified by therapeutic interventions including exposure to NPs. (Figure 1).

Current immunotherapy takes advantage of several approaches for MФ modulation, with NPs as an attractive tool. MONPs are of particular interest as they exhibit minor toxicity toward the immune cells and are able to reshape immunity both on local or systemic levels. The immunotherapeutic potential of these NPs for MФ activities and the immune system, in general, is an emerging issue (Table 1) [10,52,53,54].

## 4. Functional Outcome of Nanoparticle-Macrophage Interactions

### 4.1. External Delivery and Further Fate of Nanoparticles

**Figure 2:**
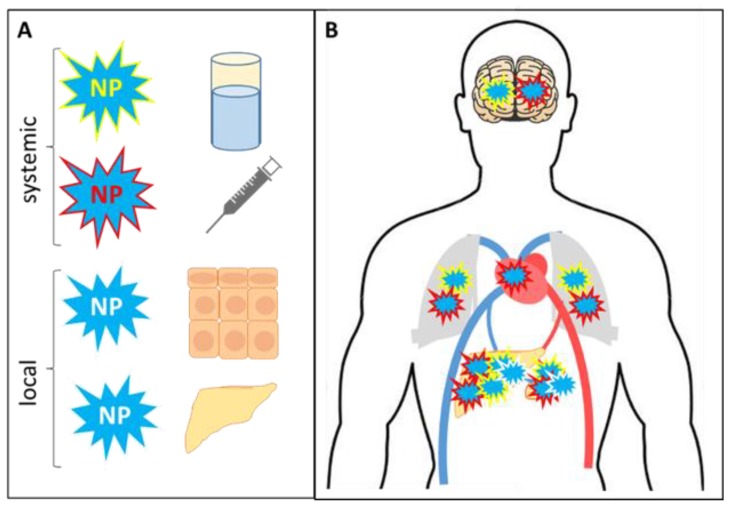
Systemic and local routes of NPs delivery and distribution. (**A**). At the systemic level, NPs can enter the organism with water/food/drug uptake or i.v. injections. Local contact with NPs occurs from skin contact, inhalation, and tumor therapy. Eventually, NPs are distributed throughout the organism in a cell free form or can be phagocytized. (**B**). When the phagocytized NPs are moving with the blood flow, they are accumulated in the heart. Air NPs primarily interact with alveolar MФs. Inhaled and injected NPs can penetrate the blood-brain barrier where they contact with microglia. The ultimate destinations of NPs are the liver and the spleen.

NPs can be delivered to the body at the systemic and/or local levels (Figure 2). To be distributed in the organism, NPs should be given with water, food or drugs, or via parenteral routes (injections). Both local and systemic NP uptake can be of an uncontrolled environmental origin; however, in this review, only therapeutic applications of nanomaterials are discussed. Locally NPs are introduced via skin contact, inhalation, or a specialized therapeutic delivery such as intraperitoneal injections when NPs are added directly to the peritoneal tumor site [74,75,76]. Once in the body, NPs are distributed freely, which is possible only in the bloodstream for a limited time, or NPs are engulfed by mononuclear cells or tissue-resident MФs (phagocytosis). Depending on the delivery route, NPs are differentially accumulated in specific organs. First of all, moving with the blood flow, either free or engulfed NPs are accumulated in the heart due to the systemic circulation, although the concentration of NPs in this organ is not the biggest. Locally delivered NPs interact with tissue-resident MФs (alveolar, skin, or others), and the majority of NPs remain within the target site. Further, the inhaled or i.v. injected NPs can penetrate the blood-brain barrier where they contact with brain resident MФs (microglia). Additionally, NPs are always found in the liver and spleen, as in these organs the life span of MФs, including those loaded with NPs, is over [77].

### 4.2. Macrophages as Nanoparticle Carriers

MФs are highly active phagocytes that can consume and/or deliver different products, including NPs, to the local inflammatory sites [61]. Also, this ’transportation’ property presents MФs as a system to deliver NPs to solid tumors that may be hardly accessible for therapeutic agents due to a dense extracellular matrix or natural barriers. Most of MONPs used in pharmacology are uptaken by MФs via clathrin-mediated endocytosis or pinocytosis and can be found within lysosomes or caveolin-1 and LAMP-1 positive endosomes [56,78,79]. Macrophages loaded with iron and tungsten oxide NPs and then injected to the tumor-bearing mice showed a significant antitumor effect in hardly accessible sites [80,81]. Thus, NP transported by MФs provide a sustainable efficacy at the local level, thereby reducing the unfavorable side effects. However, this benefit is eliminated if MФs become activated and release NPs before they reach the tumor so that careful NP design is required.

### 4.3. Regulation of Immunity

Depending on the type of metal and the biological context, MONPs can trigger pro- vs. anti-inflammatory polarization of MФs [5,82]. Activated MФs release specific cytokines and regulate the activity of neutrophils, cytotoxic, or regulatory T cells, B lymphocytes, as well as non-immune cells (fibroblasts and endothelium). Thus, the interaction of MФs with NPs controls inflammation and regeneration and represents essential immunotherapeutic tools [83]. In particular, the pro-inflammatory effect can be used in cancer therapy when activation and infiltration of immune cells correlate with better clinical prognosis [84]. In particular, the pro-inflammatory effect can be used in cancer therapy when activation and infiltration of immune cells correlate with better clinical prognosis [85]. Moreover, specific FDA approved nanoformulated drugs already showed a promising effect by converting M2-polarized tumor-associated MФs, which promote tumor survival, into M1 [54,64]. For example, carboxymethyl dextran-coated iron oxide NPs Feraheme (also called ferumoxytol) are used for drug delivery to the tumor and direct MФs towards M1 to attract cytotoxic T cells and boost up the antitumor immunity [64]. Other iron and manganese oxide NPs can also enhance antitumor immunity and suppress tumor growth and metastasis in a similar way [64,85]. Importantly, specific lymphocyte subsets are individually activated in response to NPs depending on the delivery strategy [73,86]. Some NPs, such as nickel oxide, stimulate cytokine eotaxin expression, attract neutrophils and eosinophils to the lungs and cause a severe anaphylactic reaction in mice [87].

All the above data suggest that NPs are involved in pro-inflammatory processes; however, the anti-inflammatory properties of MONPs are also being investigated. MФs treated with LPS or IFN gamma in vitro turned into M1 and showed increased activity of NFκB and STAT1 transcription factors (TFs) and higher production of IL1a, IL6, and TNF alpha. Zinc or cerium MONPs can re-direct MФs towards M2 profile, reducing the secretion of pro-inflammatory cytokines as demonstrated for primary blood monocytes and cell lines [10,60,88]. In vivo, ZnO and TiO NPs significantly reduced acute inflammation in burn wounds, pneumonia, autoimmune, and systemic LPS-driven pathologies [60,61,62,89,90]. These studies show that MONPs not only modulate the functional activity of MФs and other immune subsets but also improve tissue regeneration. One may expect that NPs enhance growth factor production by MФs, as growth factors are essential for successful recovery in these models. Another mechanism of self-protection is the reduction of MФ phagocytic activity demonstrated by iron oxide NPs in ovalbumin-sensitized mice [91]. As the wounds may be associated with hemorrhagia, it is worth noting that NPs may address the problem of bleeding when connected with thrombin; still, the immunoregulatory potential of these NPs is yet to be investigated [92]. Thus, MONPs can support pro- or anti-inflammatory activity of MФs and the immune system in total in a context- and microenvironment-specific mode. This fact reiterates the promising and complex influence of NPs on the immune status. An in-depth understanding of MФ-MONP interactions is required to fully uncover the immunotherapeutic potential of MONPs [60,61].

### 4.4. Molecular Mechanisms of Nanoparticle-Mediated Macrophage Polarization

Besides NPs phagocytosis by MФs, the interaction of NPs with MФ surface receptors may be of physiological significance. This interaction activates intracellular signaling pathways of MФs, and phagocytosis is not needed for this activation [67]. Until now, the molecular basis of NP-MФ interactions remains unclear due to the diversity of chemical composition and physical properties of NPs and to a variety of cellular contexts [93]. Some researchers classified MФs by the applied stimulus, thereby determining unlimited subsets, e.g., MФs (LPS/IL4/etc). The incentive in vivo is often undetermined, and MФs exhibit mixed M1/2 features [94]. Moreover, the inflammatory microenvironment contains a heterogeneous population of peripheral and resident MФs such as infiltrating monocytes and microglia in CNS [95,96]. Accordingly, MФs will present multiple phenotypes following the exposure to various NPs.

**Figure 3:**
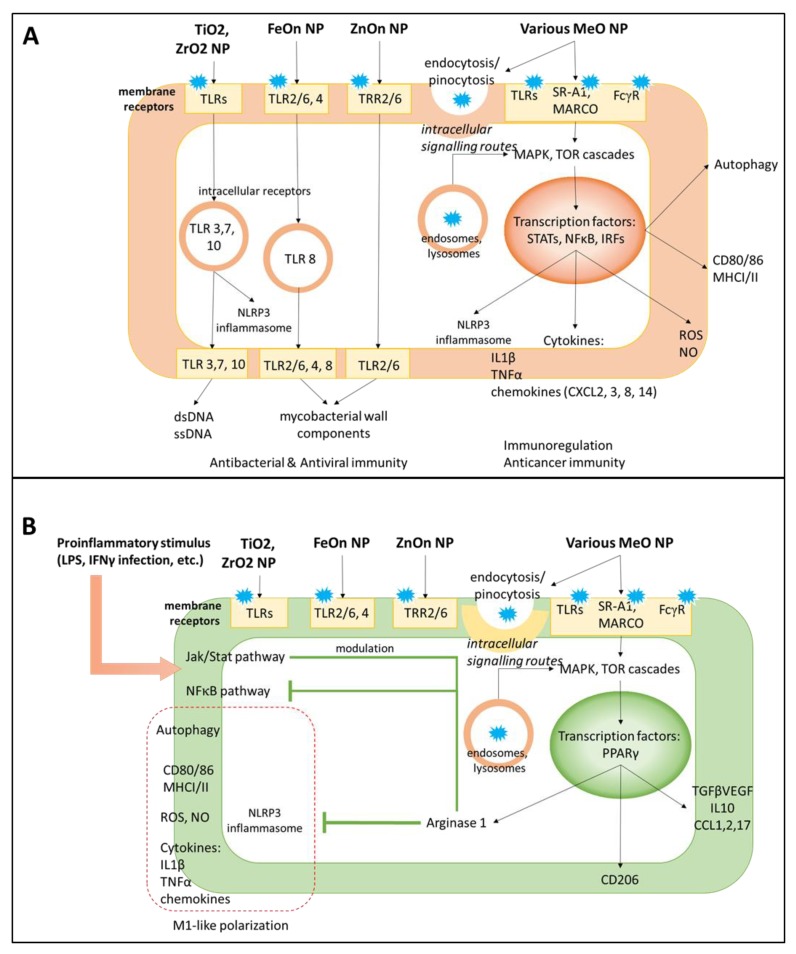
Molecular mechanisms of pro- and anti-inflammatory effects of NPs on MФs. NPs interact with cell surface receptors and can enter the cells via endocytosis/pinocytosis. (**A**). Pro-inflammatory signaling of NPs activates Toll-like (TLRs), Fcγ, and SR-A1 and MARCO scavenger receptor pathways with involved downstream MAPK/mTOR cascades and transcription factors STATs, NFkB and IRFs. NPs stimulate cytokine production and release, inflammasome formation, and phagocytic activity, thereby prompting M1 polarization. The immunostimulatory effect of NPs re-shapes the immunosuppressive microenvironment and boosts up antimicrobial or anticancer immunity. (**B**). The anti-inflammatory activity of NPs is applied to M1 committed MФs, as in chronic inflammatory disorders, autoimmunity, and neurodegeneration. NPs activate transcription factor PPARγ and arginase 1 to inhibit pro-inflammatory NFκB, modulate Jak/STAT pathway, and limit inflammation.

In general, the major pro-inflammatory potential of MONPs is mediated by Toll-like receptor (TLR) signaling [97] (Figure 3). The TLR pathway stimulation is an emerging strategy in cancer immunotherapy aimed at obtaining tumor-suppressive MФs and activation of adaptive immunity [98,99,100]. MONPs exhibit differential potency for TLRs determined by metal type. Indeed, iron oxide NPs up-regulated cytokine production in MФs via TLR2/6, 4, and 8 in a dose-dependent manner [101]. Zinc oxide NPs preferentially interacted with TLR6 in primary mouse MФs; however, other TLRs were also involved. Alternative routes for MФ-NP interactions include complement, Fcγ, and scavenger (SR-A1 and MARCO) receptor pathways. It is likely that the pro-inflammatory effect of NPs requires several cascades since the antagonists of one single receptor failed to abolish inflammation completely [101]. Scavenger receptors participate not only in MФ polarization but are responsible for NP uptake; NP phagocytosis is significantly reduced by scavenger receptor ligands heparin, fucoidan, and dextran sulfate [102].

Activation of the lysosomal autophagy system in MФs is required for NP phagocytosis. Accordingly, most NPs are positive regulators of autophagy in MФs (Figure 3). Mainly, TLR4 signaling triggered by NPs results in the upregulation of autophagy markers Sqstm and Lc combined with lysosome formation and accumulation inside the cell [103]. It is known that for iron, cerium, and titanium oxide NPs, the TF EB (TFEB), a member of the mTOR pathway, mediates autophagy of MФs [103,104,105,106,107].

Importantly, while circulating mononuclear cells have to be activated to implement their phagocytic function, the tissue-resident MФs do it routinely to eliminate damaged cells and cell debris, and thus cannot be attributed to resting or M0 [108,109]. This suggests differential activity of TFEB and diverse effects of MONPs for specific mononuclear/MФ subsets. In disease, activation of autophagy in MФs is required for bacteria or virus elimination and can reveal an additional therapeutic application of antioxidant NPs [110,111].

TLRs drive specific signaling in the immunocompetent cells, namely, stimulate the generation of reactive oxygen species (ROS), nitric oxide (NO) and inflammasome production to directly kill pathogens; increase antigen presentation as evident by up-regulated expression of major histocompatibility complex I and II (MHC I, II), CD80, CD86, deliver the cytokines and attract other subpopulations to the inflammatory site. ROS and NO participate in response to the pathogen; however, these species are also considered common markers of NP toxicity and cellular stress in general [112,113]. Zhou et al. showed that ROS production is mediated by p53 acetylation and is essential for M1-like polarization in iron-overloaded NPs; a similar mechanism can operate for MONPs [114]. Of interest, individual MONPs induce ROS of different composition and stability, with more active forms generated by TiO_2_ [115]. Downstream TLR signaling results in activation of MAPK cascades, as inhibitors of ERK, JNK, and p38 protein kinases have been shown to reduce a pro-inflammatory Il1β secretion induced by NPs [116]. Activation of these pathways results in metabolic and functional alterations in MФs including up-regulation of M1 surface marker CD86 and differential expression of pro-inflammatory cytokines (Il1β, TNFα, IL8) and chemokines (CXCL8, CXCL2 and 3, CXCL14) [64,111]. When secreted, these cytokines attract other mononuclear/MФ cells, neutrophils, as well as adaptive immune subsets (T and B lymphocytes, natural killer cells). Activation of TLR4 and autophagy pathways also led to the generation of ROS and NO as mediators of the pro-inflammatory potential in M1 MФs [117,118]. Indeed, MONPs increased ROS and NO levels in MФs [69]. On the other side, ROS and NO production induced by NPs can serve as markers of oxidative stress and cytotoxicity, as their levels correlate with the incidence of cell death [112].

An anti-inflammatory effect of MONPs generally develops as a negative regulator of the ongoing inflammation, such as in chronic inflammatory disorders or after M1 activating stimuli (Table 1) [68,119]. The more prominent potential has been reported for cerium, zinc, and copper oxide NPs that can down-regulate inflammation by targeting blood monocytes or tissue-resident MФs [8,55,56]. These NPs reduce the activity of STAT1 and NFκB and production of IL1b, IL6, TNFα, in LPS pre-treated monocytes or MФ-like cell lines (THP 1, RAW 264.7) (Figure 3). Also, i.v. injected cerium oxide NPs reduced cytokine and ROS production in a rat model of sepsis, thereby improving animal survival [120]. Furthermore, NPs can regulate local MФ populations, including the brain, liver (Kupffer cells), skin, airway MФs, by reducing their activation and pro-inflammatory cytokine secretion [8,55,58,59,62,106,121]. Interestingly, the work of Wu et al. shows that iron NPs not only mitigated cytokine expression but also attenuated cathepsin B and, thus, inhibited lysosomal secretion in microglia [121].

The effects of MONPs on MФs involve gene transcription regulation [122,123]. The TFs abundant in MФs include M1-associated STAT1 and NFκB, as well as STAT3, STAT6, or peroxisome proliferator-activated receptor-γ (PPAR-γ) that are more common for M2 cells (Figure 3) [122,124]. Pro-inflammatory stimuli LPS and IFNγ act via TLR4 to trigger STAT1 phosphorylation and up-regulate the expression of STAT1-dependent genes, and its effect is prolonged by MONPs [125,126]. In the THP1 monocytic cell line, an NFκB inhibitor attenuated Il1β production induced by TiO2 NPs [66]. The anti-inflammatory potential of NPs is also controlled via NFκB down-regulation [57]. Following TLR signaling, the TFs TFEB and Nrf2 translocate to the nucleus and positively regulate the expression of autophagy-related genes *Sqstm*1 and *LC*3 [103,127].

Gene expression analysis reveals other TFs that may respond to NP exposure in a more specific way. The activity of MФ TFs Zeb2, Smarca5, and Smarcad1 is regulated by ZnO NPs, but not other MONPs, and TFs specific for other MONPs are expected to be identified [106]. An additional mechanism of transcriptional regulation in MФs can be a Mediator complex that controls RNA polymerase II-mediated gene transcription in a highly specific and context-dependent way. Importantly, Mediator is functionally associated with major pro-inflammatory TFs STAT1 and NFκB [128]. The Mediator's kinase module consisting of Med12-13, cyclin C and CDK8/19 is involved in MФ profiling in response to NPs; however, their roles in M1/M2 polarization remain to be elucidated [106].

## 5. Therapeutic Applications of Nanoparticle-Macrophage Interactions

### 5.1. Nanoparticle-Macrophage System for in Vivo Imaging

MONPs associated with MФs can penetrate hardly accessible sites for therapeutic and diagnostic purposes. Optical properties allow MONP visualization of various tissues with enhanced contrast. Manganese and iron oxide nanoparticles were used to produce positive and negative contrast, respectively, and were tested in rats to detect the transplanted glioma cells in the brain. Moreover, MONPs can also act as a complex pH-responsible T_1_ contrast agent in cancer cells as they are sensitive for pH alterations in tumor microenvironment [129,130]. Paramagnetic gadolinium oxide nanoparticles are known to be good contrast agent for both in vivo fluorescence and magnetic resonance imaging [131]. Among the iron oxide, manganese oxide, and gadolinium oxide nanoparticles, the last ones possess the highest MR contrast possibilities [132]. In some applications, hybrid gold/iron oxide nanoparticles are reported to be advanced contrast agents for optical imaging [133].

Subsequently, the MONPs phagocytized by MФs can become essential tools for the detection and monitoring of the inflammatory sites, to which MФs are recruited [134]. Examples are experimental autoimmune encephalitis, a mouse model of multiple sclerosis with relapse-remitting course, and regular accidents of the blood-brain barrier disruption and subsequent progressive neurodegeneration. Iron oxide NPs supplemented with europium for better visualization were detected by magnetic resonance imaging, MRI in the mouse brain only during disease outbreak. Interestingly, MONPs were associated with monocyte/macrophage subset within choroid plexus and, thus, showed the damaged site of the brain and levels of neuroinflammation [135]. Another application is detection of atherosclerotic plaques [136] or lesions of pulmonary inflammation [137], where MФs are routinely present. Importantly, the imaging generally based on the optical properties of MONPs can also be supplemented by fluorescent probe labeling [136].

### 5.2. Cooperative Nanoparticle-Macrophage System Applications in Immunotherapy

The role of MONPs in immunotherapy is rapidly emerging. For instance, iron oxide NPs that have been approved by the Food and Drug Administration (FDA) modulate MФ activity and show promising results in cancer immunotherapy [65]. Since metals may exhibit both pro- or anti-inflammatory effects in a context-dependent but hardly controllable way, NPs are often loaded with particular cytokines to control MФ profiles. For example, TiO2 NPs can trigger either M1 or M2 polarization of MФs. However, the effect is strictly anti-inflammatory when IL4 is added to the system. Moreover, this combination allows turning M1 into M2 MФs even at the late stage of activation, which is extremely challenging in other systems [138]. Rather than cytokines, NPs used in cancer therapy can carry tumor antigens to activate MФs and CD4+/CD8+ cytotoxic T cells against the tumor. To further enhance the therapeutic effect, NPs can be loaded with small interfering RNAs to modulate the immune response or inhibit cancer cell proliferation and survival [139].

Of particular interest are MONPs that can attenuate some side effects of chemotherapy. Indeed, doxorubicin drives M2 MФ polarization, thereby increasing the risk of neovascularization, growth factor release, and tumor survival. However, MФs turn into M1, as shown by the increased TNFα production, when loaded with doxorubicin + ZnO NPs [140]. Finally, NPs can significantly improve drug delivery towards solid tumors or metastatic lesions, including CNS, and represent a smart delivery system for precise and efficient immunotherapy [141].

Beyond cancer immunotherapy, NPs may control MФ polarization for wound healing in trauma or diabetes [61,142]. There is also an increasing need for NPs tailor-suited for both diagnostic and therapeutic applications (see also *Nanoparticle-macrophage system for in vivo imaging*). One example comes from gold iron oxide NPs coupled with an anti-CD163 antibody for MRI detection of activated MФs in atherosclerotic lesions or inflamed kidneys and, in perspective, for selective control of MФ subsets [143,144].

Nevertheless, cytotoxicity remains a major concern in the manufacturing and therapeutic applications of NPs and have to be addressed in the future [5]. Toxicity depends on the metal type, structural properties, and the dose of exposure [9,145,146,147]. The conventional approach to eliminate cytotoxicity is to combine different metals to maximize the desired therapeutic effect while minimizing off-target oxidative stress and cell death. For example, a combination of copper with ZnO NPs reduces apoptosis in MФs RAW264.7 [148]. Moreover, a combination of different metal ions may help to control MФ activation and inflammation [149]. Magnesium added to TiO_2_ down-regulated the expression of pro-inflammatory markers TNFα, IL6, and IL1β and up-regulated the anti-inflammatory CD163 in LPS-primed MФs [150]. TiO_2_ NPs doped with Ag evoked more pronounced toxicity towards the tumor, but not non-malignant cells, compared to TiO_2_ alone [151]. The potency of NPs against tumor cells helps to overcome a non-specific activity of NPs in the sites of accumulation [113] (Figure 2).

## 6. Future Directions and Conclusions

MONPs as drugs alone or drug carriers have proved their efficacy in a variety of biomedical applications. A new attractive area that goes beyond these situations is a cooperation between MONPs and their cellular hosts. MФs, whose primary biological function is phagocytosis, are a perfect target for exogenous nanobiomaterials. This immanent property is advantageous for engineering MФs with various MONPs to produce a controlled tool for powerful immunoregulation at local and systemic levels and is of particular importance for delivery to hardly accessible sites as CNS or tumor [81]. MONPs are expected to properly polarize MФ into a pro- or anti-inflammatory phenotype to optimize the immune function for the antitumor response, prevention of autoimmunity, and control of tissue architectonics. Given that MФs loaded with magnetic NPs can be visualized and localized to the desired site using external equipment, MФs-MONPs paradigm emerges as a novel strategy for immunotherapeutic interventions in disease.

## Figures and Tables

**Table 1:** Effects of MONPs on pro- and anti-inflammatory activities of МФs.

Macrophage Cells/	Functional Effect of Nanoparticles	Reference
In Vivo Model		
**Cerium Oxide NPs**		
Kupffer cells and peripheral macrophages from LPS-treated mice	Reduced NFκB TF activity, cytokine and ROS release, reduced inflammation	Selvarai et al., 2015 [55]
RAW 264.7 following oxidative stress	Reduced ROS release	Xia et al., 2008 [56]
Rat model of liver fibrosis	Reduced MФ activation and cytokine release	Oro et al., 2016 [8]
**Zinc oxide NPs**		
RAW 264.7 stimulated with LPS and IFNγ	Reduce NFκB TF activity, Il1β, and TNFa release	Kim & Jeong, 2015 [57]
Blood mononuclear cells stimulated with LPS	Reduced Il1β and IL6 production. Activation of eIF2, eIF4 and mTOR pathways	Makumire et al., 2014 [58]
Alveolar macrophages from infected mice (influenza)	Decreased NFκB activation and NO release, suppressed bacterial clearance	Lin et al., 2014 [59]
	Reduced oxidative stress: aromatase expression, glutathione peroxidase, and reductase activity	
Burn wounds	Improved anti-microbial activity and wound healing;	Ali et al., 2017 [60]
	inhibited albumin denaturation and proteinase activity	Seisenbaeva et al., 2017 [61]
Atopic dermatitis	Decreased F4/80+ macrophage infiltration, reduce pro-inflammatory cytokines	Ilves et al., 2014 [62]
Rats after ZnO exposure;	Activate microglia via NFκB, ERK, and p38 and stimulate neuroinflammation	Liang et al., 2018 [63]
BV2 microglial cell line		
Peripheral blood mononuclear cells;	Increase IFN, TNFΑ, and IL12. Induce ROS production, oxidative stress, and inflammation	Xia et al., 2008 [56]
RAW 264.7		
**Iron oxide NPs**		
RAW 264.7 macrophages alone or with cancer cells; adenocarcinoma mouse model	Up-regulate M1 markers (TNFa, CD86) and ROS;	Zanganeh et al., 2016 [64]
Melanoma mouse model	activate Th1 response and anticancer immunity; reduce tumor growth	Luo et al., 2019 [65]
	induce activation of macrophages and T cells and maturation of dendritic cells	
**Titanium oxide NPs**		
THP1 macrophages	Increase Il1β and inflammasome production in NFκB dependent mode	Fukatsu et al., 2018 [66]
THP1; mouse bone marrow-derived MФs;	Inflammasome formation, Il1β and a release; lung inflammation	Yazdi et al., 2010 [67]
Pulmonary inflammation		
Myelomonocytic U-937 cells	Increased TLR3,7,10; no effect on cytokines	Lucarelli et al., 2004 [68]
CNS inflammation	Increased ROS and NO production	Wu and Tang, 2017 [69]
THP1 macrophages	Polarize towards M2 (up-regulate arginase 1, mannose receptor, IL10) via PI3K/Akt and Erk1/2	Xu et al., 2019 [70]
**Copper oxide NPs**		
LPS-treated RAW 264.7 and mouse bone marrow-derived MФs	Inhibit phagocytosis, reduce NO production	Triboulet et al., 2013 [71]
Mouse peritonitis model	Recruit MФs	Arancibia et al., 2016 [72]
LPS-primed peritoneal MФs	reduce NO production in an arginase dependent model	
Myelomonocytic U-937 cells	Inhibit CD14 expression, induce TNFα, reduce IL1Rα	Lucarelli et al., 2004 [68]
**Lanthanum oxide NPs**		
Inhalation	Acute airway inflammation	Sisler et al., 2017 [9]
**Cobalt oxide NPs**		
Peripheral macrophages	Increase IFNγ and TNFα, attract CD4+ cells	Chattopadhyay et al., 2013 [73]

MФ polarization is determined by NP types and conditions in the surrounding microenvironment. The evidence on pro- or anti-inflammatory effects of NPs are highlighted in red and green, respectively.

## Abbreviations

IL	Interleukin
IFNγ	Interferon gamma
LPS	Lipopolysaccharide
MO	Metal oxide
MФ	Macrophage
MRI	Magnetic resonance imaging
NO	Nitric oxide
NP	Nanoparticle
ROS	Reactive oxygen species
TF	Transcription factor
TLR	Toll-like receptor
